# Psychometric Properties of the Italian Food Noise Questionnaire (FNQ) for the Assessment of Intrusive Food-Related Thoughts

**DOI:** 10.3390/bs16040609

**Published:** 2026-04-20

**Authors:** Edoardo Mocini, Olivia Di Vincenzo, Clarissa D’angelo, Carlo Baldari, Silvia Migliaccio, Andrea Zagaria

**Affiliations:** 1 Department of Theoretical and Applied Sciences, eCampus University, 22060 Novedrate, Italy; 2 Department of Experimental Medicine, Sapienza University of Rome, 00161 Rome, Italy; 3 Independent Researcher, 00179 Rome, Italy; 4 Department of Systems Medicine, Tor Vergata University of Rome, 00133 Rome, Italy; andrea.zagaria@uniroma2.it

**Keywords:** food noise, intrusive thoughts, eating behavior, psychometric properties, obesity, food preoccupation, perseverative thinking, disordered eating

## Abstract

Food noise refers to persistent and intrusive thoughts about food that may interfere with daily functioning, emotional well-being, and eating behaviors. Although the construct has gained increasing attention in clinical and research contexts, no psychometrically sound tools are currently available in Italian to assess food noise. Therefore, the present study aimed to translate, adapt, and evaluate the psychometric properties of the Italian version of the Food Noise Questionnaire (FNQ). A total of 1087 participants (mean age 37.45 ± 10.35 years; 50.6% female) were enrolled in the investigation. Participants completed the Italian version of the FNQ, along with a convergent measure of food-related preoccupation and self-report measures of depressive symptoms, anxiety symptoms, and perceived stress. Confirmatory factor analysis supported a unidimensional factor structure for the FNQ, with salient standardized factor loadings (range = 0.803–0.919) and strong internal-consistency reliability (categorical ω = 0.917). Evidence of convergent validity was provided by strong associations with food-related preoccupation (*r* = 0.831, *p* < 0.001), whereas discriminant validity was supported by smaller, yet significant, correlations with anxiety, depression, and perceived stress (*r* range = 0.350 to 0.417, *p* < 0.001). In addition, configural, metric, and scalar invariance across gender was established within a multi-group framework. These findings provide evidence for the FNQ as a reliable and valid measure of food noise in the Italian adult population, demonstrating robust psychometric properties and gender-invariant measurement.

## 1. Introduction

Obesity is a multifactorial chronic condition characterized by an excess of adipose tissue, in which cognitive and emotional aspects of eating behavior play a significant role, including persistent and intrusive thoughts about food that can impact daily functioning and weight management (Heymsfield & Wadden, 2017). Food noise has been defined as repetitive, unwanted food-related mental activity that may interfere with attention, mood, and eating behavior (Diktas et al., 2025; Hayashi et al., 2023). This construct has recently been conceptualized within the broader framework of food cue reactivity, and proposed as a distinct, measurable phenomenon (Boswell & Kober, 2016; Hayashi et al., 2023; Kanoski & Boutelle, 2022). Within the broader literature on eating-related constructs, food noise shows partial overlap with, but remains conceptually distinct from, constructs such as food craving, food preoccupation, and attentional bias toward food-related stimuli. While food craving typically refers to the motivational and affective desire to consume specific foods (Cepeda-Benito et al., 2000), and food preoccupation captures the frequency and salience of food-related thoughts (Tapper & Pothos, 2010), food noise emphasizes the intrusive, repetitive, and often uncontrollable nature of such cognitions, as well as their perceived impact on daily functioning. Moreover, these cognitive processes can be situated within broader frameworks of food cue reactivity and cognitive load, which have been linked to dysregulated eating behaviors and increased attentional capture by food-related stimuli (Boswell & Kober, 2016; Higgs, 2015). In addition, emerging evidence suggests that eating-related cognitions may interact with higher-order psychological constructs such as internalized weight stigma, which has been associated with psychological distress, disordered eating, and reduced quality of life (e.g., Zagaria et al., 2023). From this perspective, food noise may represent a specific cognitive mechanism at the intersection of cue reactivity, perseverative cognition, and affective vulnerability.

In the context of health psychology, increasing attention has been devoted to cognitive processes that mediate the relationship between environmental food exposure, internal states, and eating behavior. Contemporary food environments are characterized by the constant availability and salience of highly palatable foods, which may promote persistent cognitive engagement with food-related cues (Hayashi et al., 2023; Kanoski & Boutelle, 2022; Swinburn et al., 2011). From this perspective, intrusive food-related thoughts can be conceptualized as a psychological mechanism through which environmental stimuli interact with individual vulnerability factors, such as reward sensitivity, emotion regulation difficulties, and learned eating patterns (May et al., 2010; Tapper & Pothos, 2010). These cognitive processes may contribute to maladaptive eating behaviors not only by influencing food intake directly, but also by increasing psychological burden, attentional capture, and emotional distress in daily life (Boswell & Kober, 2016; Higgs, 2015; Ward & Mann, 2000).

Despite its growing relevance, food noise has only recently been operationalized through the development of the Food Noise Questionnaire (FNQ; Diktas et al., 2025), a brief five-item self-report measure assessing the frequency and impact of intrusive food-related thoughts. In the original validation study (Diktas et al., 2025), the FNQ demonstrated a unidimensional structure, high internal consistency (Cronbach’s α = 0.93), test–retest reliability over a 7-day interval (*r* = 0.79), as well as evidence of convergent validity with a convergent measure of food-related preoccupation and discriminant validity with respect to stress, anxiety, and depressive symptoms.

However, to the best of our knowledge, no Italian version of the FNQ is currently available. Given the growing scientific relevance of the food noise construct and the lack of a measure in Italian-speaking populations, the present study aimed to translate, culturally adapt, and examine the psychometric properties of the FNQ in a general Italian adult sample. Specifically, the study aimed to examine the dimensionality of the scale, hypothesizing a unidimensional structure in line with Diktas et al. (2025), as well as its internal consistency and convergent and discriminant validity. With regard to construct validity, it was expected that the pattern observed in the original validation study would be replicated (Diktas et al., 2025), with strong correlations with a convergent measure of food-related preoccupation and smaller, yet significant, correlations with anxiety, stress, and depressive symptoms.

## 2. Materials and Methods

### 2.1. Study Design and Procedure

The present study is part of a larger observational research project aimed at investigating subjective cognitive experiences related to eating behavior in the Italian adult population, with a specific focus on intrusive food-related thoughts.

A cross-sectional design was adopted, and data were collected through an anonymous, online survey hosted on Google Form platform. Participants were recruited via social media platforms, and participation was voluntary with no incentives. Data were collected between 1 October and 30 November 2025. Prior to accessing the online survey, participants provided electronic informed consent after being informed about the study aims, procedures, data handling, and anonymity. The survey required approximately 10 min to complete. Attention check items (e.g., simple factual questions such as “What is the capital of Italy?”) were included to identify careless responding.

The study protocol was approved by the Ethics Committee of eCampus University (approval number 13/2025), and all procedures were conducted in accordance with the Declaration of Helsinki.

### 2.2. Participants

Inclusion criteria were being 18 years of age or older, being a native Italian speaker, and providing informed consent; no exclusion criteria were applied. Sample size determination followed two criteria. First, to evaluate the dimensionality and internal consistency of the scale, a minimum ratio of 10 participants per item was adopted, corresponding to a minimum sample size of 50 participants (De Vet et al., 2011). Second, an a priori power analysis suggested that 193 participants were required to detect a practically significant correlation (ρ = 0.20) with 80% power at a significance level of α = 0.05 (Ferguson, 2009).

A total of 1087 participants were thus enrolled. Of these, 49.4% were male and 50.6% were female. The mean age was 37.45 years (SD = 10.35), with an age range of 18 to 78 years. Regarding educational level, 23.6% of participants had completed high school, 54.6% held a university degree, 20.1% had completed postgraduate studies, and 1.7% had a middle-school diploma. In terms of relationship status, 39.4% reported being in a relationship, 28.2% were married, 28.2% were single, 3.9% were divorced, and 0.4% were widowed. The mean body mass index (BMI) was 25.24 kg/m^2^ (SD = 5.43).

### 2.3. Measures

#### 2.3.1. Socio-Demographic Variables

Participants reported socio-demographic information using a standardised form, including age, gender, education, relationship status and self-reported height and weight.

#### 2.3.2. Italian Food Noise Questionnaire (FNQ)

Food noise was assessed using the FNQ (Diktas et al., 2025). The scale consists of five items designed to assess intrusive food-related thoughts. Items are rated on a five-point Likert scale ranging from 0 (strongly disagree) to 4 (strongly agree). The questionnaire instructions were as follows: “*Please answer these questions by reflecting on your thoughts over the last 2 weeks only*.” A total score is obtained by summing item responses, with higher scores indicating greater levels of food noise.

The translation process followed established guidelines for the cross-cultural adaptation of self-report and patient-reported outcome instruments (i.e., forward and back-translation procedure) (Beaton et al., 2000; Wild et al., 2005; Kulis et al., 2011; Zagaria et al., 2022). First, two Italian researchers with expertise in the target construct and in psychological assessment independently translated the original English version of the FNQ into Italian. The two translations were then compared and reconciled into a single Italian version. This version was subsequently back-translated into English by an English-speaking researcher who was blinded to the original version in order to identify and resolve conceptual inconsistencies. Finally, the Italian version was pilot-tested in a sample of 15 participants from the target population to assess the clarity of the items; no issues were reported, and no further revisions were required.

The Italian version of the FNQ can be requested by emailing the corresponding author.

#### 2.3.3. Food Cravings Questionnaire—Trait (FCQ-T)

Trait-level food craving was assessed using the Food Cravings Questionnaire–Trait (FCQ-T) (Cepeda-Benito et al., 2000). Specifically, the Thoughts subscale was administered in order to capture thoughts and preoccupation with food and to provide evidence for the convergent validity of the FNQ (Innamorati et al., 2014). This subscale consists of 7 items, rated on a 6-point Likert scale ranging from never (1) to always (6) (e.g., “I find myself preoccupied with food”). Omega coefficient in the present sample was 0.939, highlighting excellent internal consistency.

### 2.4. Psychological Distress

Symptoms of anxiety and depression were assessed using the Generalized Anxiety Disorder–7 (GAD-7) (Shevlin et al., 2022) and the Patient Health Questionnaire–9 (PHQ-9) (Shevlin et al., 2022), respectively. The GAD-7 is a seven-item self-report questionnaire assessing generalized anxiety symptoms experienced over the past two weeks, with items rated on a 4-point Likert scale ranging from 0 (not at all) to 3 (nearly every day). The PHQ-9 is a nine-item self-report measure assessing depressive symptoms over the past two weeks, using the same 4-point response format. Omega coefficients in the present sample were 0.896 and 0.862 for GAD-7 and PHQ-9, respectively, indicating strong internal consistency.

Perceived stress was assessed using the Perceived Stress Scale (PSS) (Mondo et al., 2021), a 10-item self-report instrument that assesses the extent to which situations in one’s life are appraised as stressful. Items are rated on a 5-point Likert scale ranging from 0 (never) to 4 (very often). Omega coefficient in the present sample was 0.894, suggesting good internal consistency.

### 2.5. Data Analyses

Data were analyzed using *jamovi* (version 2.6.26) and the R package *lavaan* (Rosseel, 2012). First, descriptive statistics for each item of the FNQ were computed, including mean, standard deviation, skewness, and kurtosis. To determine the most appropriate estimator for the confirmatory factor analysis (CFA), the distributional properties of the items were examined. Specifically, skewness and kurtosis values within the range of −1 to +1 were considered indicative of negligible deviations from univariate normality (Muthén & Kaplan, 1985).

The factor structure of the FNQ was subsequently examined through CFA. Consistent with the original structure proposed by Diktas and colleagues (2025), a unidimensional measurement model was tested. Model fit was evaluated using multiple goodness-of-fit indices (Wang & Wang, 2019): the Comparative Fit Index (CFI) and Tucker–Lewis Index (TLI), with values ≥ 0.90 indicating acceptable fit; the Standardized Root Mean Square Residual (SRMR), with values ≤ 0.08 indicating acceptable fit; and the Root Mean Square Error of Approximation (RMSEA), with values ≤ 0.08 indicating acceptable fit.

Measurement invariance across gender was further assessed using multi-group CFA (MG-CFA) via the stepwise procedure proposed by Meredith (Meredith, 1993), i.e., by sequentially testing nested models including configural invariance (same pattern of free and fixed factor loadings across groups), metric invariance (equality of factor loadings across groups), and scalar invariance (equality of item thresholds across groups). The adequacy of invariance constraints was evaluated by comparing nested models and examining changes in fit indices. Following established guidelines (Cheung & Rensvold, 2002; Chen, 2007), decreases in CFI greater than 0.01 and increases in RMSEA greater than 0.015 were considered indicative of a lack of invariance. All CFA models were estimated using the diagonally weighted least squares (DWLS) estimator with a polychoric correlation matrix, which is recommended for ordinal response data (e.g., Li, 2016).

Internal consistency reliability of the FNQ was assessed using omega coefficients designed for ordered categorical indicators (Green & Yang, 2009), which is considered more robust than Cronbach’s alpha due to less restrictive measurement assumptions, e.g., omega does not require essentially tau-equivalence (Flora, 2020). Omega values ≥ 0.70 were interpreted as evidence of satisfactory internal consistency (Hair et al., 2019).

Finally, construct validity of the scale was examined by calculating zero-order correlation coefficients between FNQ scores and the Thoughts subscale of the FCQ-T, as well as with measures of depressive symptoms (PHQ-9), anxiety symptoms (GAD-7), and perceived stress (PSS). Evidence of convergent and discriminant validity was inferred based on the magnitude and direction of these associations.

## 3. Results

### 3.1. Item-Level Descriptive Statistics

Item-level descriptive statistics are reported in Table 1. All response categories of the Likert-type scale were endorsed at least once (range = 0–4). Item means ranged from 1.137 to 1.779. Skewness and kurtosis values ranged from −1.048 to 0.772, indicating negligible departures from univariate normality, as most values fell within the ±1 range (Muthén & Kaplan, 1985). Given that item responses are scored on an ordered categorical scale and considering their distributional properties, the diagonally weighted least squares (DWLS) estimator was employed for the subsequent CFAs using a polychoric correlation matrix (Li, 2016).

### 3.2. Factor Structure

A one-factor CFA model was specified, showing an overall acceptable fit to the data: χ^2^(5) = 58.509 (*p* < 0.001), RMSEA = 0.099, CFI = 0.998, TLI = 0.996, and SRMR = 0.035. Although the RMSEA value exceeded conventional cut-offs (Wang & Wang, 2019), indicating suboptimal fit, this index is highly sensitive to model degrees of freedom (df) and may overstate misfit when df are low (Kenny et al., 2015; Shi et al., 2022). As noted by Kenny et al. (2015, p. 503), “using the RMSEA to assess the model fit in models with small df is problematic and potentially misleading” and researchers are advised “not to dismiss models with large RMSEA values with small df without examining other information”; accordingly, RMSEA should be interpreted cautiously in the present model where *df* = 5 (Kenny et al., 2015; Taasoobshirazi & Wang, 2016; Shi et al., 2022). On the other hand, the incremental fit indices (CFI, TLI) and the residual-based index (SRMR) indicated an excellent fit to the data, thus supporting the unidimensional solution (Hu & Bentler, 1999).

Importantly, the standardized factor loadings of the CFA model were uniformly high, ranging from 0.803 to 0.919 (M = 0.864, SD = 0.050; *p* < 0.001) and attesting to a substantial proportion of common variance among the indicators. The average variance extracted (AVE) was 0.748, indicating that more than half of the variance in the indicators was accounted for by the construct, exceeding the recommended threshold of 0.50 (Hair et al., 2019).

**Figure 1:**
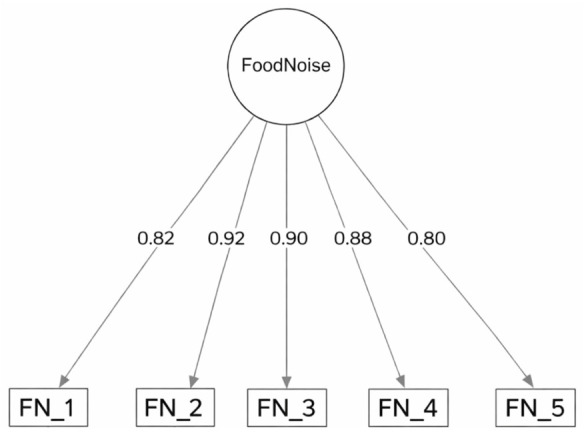
Completely standardized factor loadings from the one-factor confirmatory factor analysis (CFA) model. All factor loadings were statistically significant (*p* < 0.001). Abbreviation: FN = food noise.

Detailed results are presented in Figure 1.

### 3.3. Factorial Invariance Tests Across Gender

Factorial invariance tests across gender (males vs. females) were carried out using multiple-group confirmatory factor analysis (MG-CFA). The one-factor model examined simultaneously in males and females (i.e., configural invariance), without imposing any constraints, showed an acceptable overall fit to the data: χ^2^ (10) = 63.546 (*p* < 0.001), RMSEA = 0.099, CFI = 0.998, TLI = 0.996, SRMR = 0.038. Afterwards, equality constraints were imposed on factor loadings and item thresholds, and no meaningful decline in model fit was observed (ΔCFI < 0.010 and ΔRMSEA < 0.015), supporting both metric and scalar invariance models across gender, respectively. Detailed results are reported in Table 2.

### 3.4. Reliability and Validity

The categorical omega coefficient was 0.917, indicating excellent internal consistency reliability of the FNQ total score. Regarding construct validity, convergent validity was supported by an expected large correlation between FNQ and the thoughts subscale of the FCQ-T (*r* = 0.831, *p* < 0.001). By contrast, discriminant validity was supported by moderate correlations between FNQ and symptoms of anxiety, depression, and perceived stress, as measured by the GAD-7 (*r* = 0.350, *p* < 0.001), PHQ-9 (*r* = 0.417, *p* < 0.001), and PSS (*r* = 0.376, *p* < 0.001), respectively. Lastly, a moderate positive correlation was observed between FNQ scores and BMI (*r* = 0.310, *p* < 0.001).

Descriptive statistics of the psychometric measures are reported in Table 3.

## 4. Discussion

The present study aimed to translate, culturally adapt, and examine the psychometric properties of the Italian version of the FNQ among an adult sample. Overall, the findings support the FNQ as a reliable tool for assessing intrusive food-related thoughts, highlighting a unidimensional structure, strong internal consistency, and theory-consistent associations with convergent measures of food-related preoccupation, as well as anxiety, depression and perceived stress symptom scores.

More specifically, confirmatory factor analysis supported a one-factor structure, consistent with the original conceptualization of the FNQ (Diktas et al., 2025). All items displayed substantial standardized factor loadings, indicating that they contributed meaningfully to a single latent dimension (range 0.80 to 0.92). Importantly, MG-CFA across gender supported configural, metric, and scalar invariance models. These results indicate that the Italian version of the FNQ has the same pattern of free and fixed loadings in males and females and that factor loadings and item thresholds are comparable across gender groups. These results allow meaningful gender-based comparisons of FNQ scores in clinical and research settings, such as latent mean comparisons.

Crucially, the Italian version of the FNQ showed excellent internal consistency (categorical ω = 0.917), suggesting that approximately 92% of the variance in the total score is attributable to true score variance (Flora, 2020). Evidence for construct validity was obtained through bivariate Pearson’s correlation coefficients. Specifically, convergent validity was supported by a strong association between the FNQ and the Thoughts subscale of the FCQ-T (*r* = 0.83), which assesses a closely related construct of preoccupation with food (Innamorati et al., 2014). The strength of this association indicates that approximately 69% of the variance was shared between the two measures, suggesting a close interrelationship. In contrast, associations between the FNQ and measures of anxiety, depressive symptoms, and perceived stress were smaller in magnitude, yet statistically significant. This pattern is consistent with theoretical expectations and aligns with the original validation study (Diktas et al., 2025), supporting the discriminant validity of the FNQ and indicating that food noise, while related to general psychological distress, represents a distinct construct. Furthermore, the moderate association observed between FNQ and BMI is consistent with evidence showing that food noise is commonly reported among individuals with obesity, with up to 57% endorsing such experiences, thus supporting a plausible positive association (Dhurandhar et al., 2025).

From a broader perspective, these findings contribute to the growing literature on the cognitive dimensions of eating behavior. The availability of a validated Italian instrument will allow for more systematic investigation of food noise in both clinical and non-clinical populations. In particular, the FNQ may be useful for exploring the role of intrusive food-related thoughts in conditions characterized by dysregulated eating, obesity, or heightened food cue reactivity, as well as for evaluating changes associated with psychological or pharmacological interventions. Food noise may be conceptualized as an index of sustained cognitive engagement with food-related cues, reflecting heightened food cue reactivity and increased cognitive load in everyday environments (Boswell & Kober, 2016; Hayashi et al., 2023; Kanoski & Boutelle, 2022; Ward & Mann, 2000; Higgs, 2015). A growing body of literature indicates that enhanced food cue reactivity and attentional capture by food-related stimuli are associated with dysregulated eating patterns, greater subjective hunger, emotional distress, and difficulties in self-regulation, independently of body weight or the presence of formal eating disorder diagnoses (Boswell & Kober, 2016; Ward & Mann, 2000; Higgs, 2015). Persistent intrusive food-related thoughts may therefore represent a clinically relevant source of cognitive and emotional burden, with potential implications for daily functioning, quality of life, and adherence to health-related behavior change (May et al., 2010; Tapper & Pothos, 2010). In this context, the availability of the FNQ may be particularly useful for identifying individuals experiencing elevated food-related cognitive load and for monitoring changes associated with psychological interventions targeting eating-related cognitions and emotion regulation, as well as pharmacological treatments known to reduce food cue responsivity and appetitive drive (Diktas et al., 2025; Ten Kulve et al., 2016). Further, the FNQ might become a useful tool to help clinicians, who daily work with subjects affected by overweight and obesity, to better individualize and target a pharmacological or non-pharmacological therapeutical intervention.

To conclude, several limitations of the present study should be acknowledged. First, the present study employed cross-sectional design; thus, the temporal stability of FNQ scores could not be assessed and test–retest reliability remains to be explored. Second, participants were recruited online, which may have introduced selection biases and limited the representativeness of the sample. Replication in more diverse and representative populations is therefore warranted. Finally, the present study enrolled a non-clinical population; further studies are needed to examine the psychometric properties of the FNQ in clinical samples, such as patients with a formal diagnosis of eating disorders.

## 5. Conclusions

The FNQ demonstrated overall satisfactory psychometric properties, including a strong unidimensional structure, excellent reliability, and evidence of convergent and discriminant validity. Measurement invariance across gender was further established.

The Italian version of the scale represents a brief and reliable tool for assessing intrusive food-related thoughts in Italian-speaking populations. Its use may facilitate research on the cognitive mechanisms underlying eating behavior and support clinical assessment in contexts where food-related cognitive intrusions play a relevant role. Further research is warranted to explore its longitudinal psychometric properties (i.e., test–retest reliability) and clinical utility.

## Figures and Tables

**Table 1:** Item-level descriptive statistics. Abbreviation: SD, standard deviation.

	Mean (SD)	Range	Skewness	Kurtosis
1. I find myself constantly thinking about food throughout the day	1.779 (1.071)	0–4	0.236	−0.643
2. My thoughts about food feel uncontrollable	1.268 (1.087)	0–4	0.665	−0.307
3. I spend too much time thinking about food	1.579 (1.207)	0–4	0.351	−0.970
4. My thoughts about food have negative effects on me and/or my life	1.477 (1.270)	0–4	0.426	−1.048
5. My thoughts about food distract me from what I need to do	1.137 (1.069)	0–4	0.772	−0.201

**Table 2:** Factorial invariance tests across gender. Note: Models were estimated using diagonally weighted least squares (DWLS).

Model	χ^2^ (df)	CFI	TLI	SRMR	RMSEA	Model Comparison	ΔRMSEA	ΔCFI
1. Configural invariance	63.546 (10)	0.998	0.996	0.038	0.099			
2. Metric invariance	81.583 (14)	0.997	0.996	0.044	0.094	2 vs. 1	0.005	−0.001
3. Scalar invariance	85.064 (28)	0.998	0.998	0.038	0.061	3 vs. 2	−0.033	0.001

**Table 3:** Descriptive statistics (mean, standard deviation, range, skewness, and kurtosis) for Food Noise (FNQ), food-related preoccupation (FCQ-T), depressive symptoms (PHQ-9), anxiety symptoms (GAD-7), and perceived stress (PSS). Abbreviations: FNQ = Food Noise Questionnaire; FCQ-T = Food Craving Questionnaire (Thoughts subscale); PHQ-9 = Patient Health Questionnaire–9; GAD-7 = Generalized Anxiety Disorder–7; PSS = Perceived Stress Scale; SD = Standard Deviation.

Variable	Mean (SD)	Range	Skewness	Kurtosis
Food Noise (FNQ)	7.24 (4.91)	0–20	0.52	−0.51
Food-related preoccupation (FCQ-T)	15.25 (7.52)	7–42	1.15	0.81
Depressive symptoms (PHQ-9)	8.41 (5.23)	0–27	0.93	0.64
Anxiety symptoms (GAD-7)	8.21 (4.88)	0–21	0.74	−0.22
Perceived stress (PSS)	19.01 (6.98)	1–38	0.12	−0.40

## Data Availability

Data supporting reported results can be obtained upon request.

## Abbreviations

The following abbreviations are used in this manuscript:

FNQ	Food Noise Questionnaire
BMI	Body Mass Index
CFA	Confirmatory Factor Analysis
MG-CFA	Multiple Group Confirmatory Factor Analysis
DWLS	Diagonally Weighted Least Squares
CFI	Comparative Fit Index
TLI	Tucker–Lewis Index
SRMR	Standardized Root Mean Square Residual
RMSEA	Root Mean Square Error of Approximation
FCQ-T	Food Cravings Questionnaire Trait
GAD-7	Generalized Anxiety Disorder 7
PHQ-9	Patient Health Questionnaire 9
PSS	Perceived Stress Scale
SD	Standard Deviation
